# Development and external validation of a short prognostic screening instrument for PTSD one year following individual civilian trauma

**DOI:** 10.1080/20008066.2025.2594266

**Published:** 2025-12-15

**Authors:** Jeanet F. Karchoud, Chris M. Hoeboer, Nicole van Gelder, Joanne Mouthaan, Marit Sijbrandij, Miranda Olff, Rens van de Schoot, Mirjam van Zuiden

**Affiliations:** a Amsterdam UMC, University of Amsterdam, Psychiatry, Amsterdam Public Health, Amsterdam, The Netherlands; b Victim Support Netherlands, Utrecht, The Netherlands; c Department of Clinical Psychology, Leiden University, Leiden, The Netherlands; d Department of Clinical, Neuro- and Developmental Psychology, WHO Collaborating Center for Research and Dissemination of Psychological Interventions, Amsterdam Public Health Research Institute, Vrije Universiteit Amsterdam, Amsterdam, The Netherlands; e ARQ National Psychotrauma Centre, Diemen, The Netherlands; f Department of Methods and Statistics, Utrecht University, Utrecht, The Netherlands; g Department of Clinical Psychology, Utrecht University, Utrecht, The Netherlands

**Keywords:** Posttraumatic stress disorder (PTSD), trauma, longitudinal, machine learning, sex, prediction, social support, posttraumatic cognitions, peritraumatic dissociation, peritraumatic distress, Trastorno de estrés postraumático (TEPT), trauma, longitudinal, aprendizaje automático, sexo, predicción, apoyo social, cogniciones postraumáticas, disociación peritraumática, malestar peritraumático

## Abstract

**Background:** Timely identification of individuals at risk for developing PTSD following trauma is crucial for providing targeted preventive interventions. Machine learning techniques show promise for deriving accurate prognostic screening instruments. However, accurate externally validated prognostic screening instruments for broad application in trauma-exposed civilians are not yet available. Moreover, it remains unknown whether prognostic screening instrument accuracy may be improved if developed in a sex-stratified manner.

**Objective:** We aimed to develop an externally validated prognostic PTSD screening instrument based on self-report information obtained within 2 months post-trauma in two independent cohorts of recently trauma-exposed civilians, using machine learning techniques allowing for extraction of a short screener. We examined whether separate models for males and females improved prognostic accuracy compared to sex-combined models.

**Methods:** Prognostic machine learning models (CART and XGBoost) were developed in a longitudinal cohort of *N* = 327 adults (38% females) requiring evaluation of (suspected) serious injury by an emergency department. External validation was performed in another longitudinal cohort of *N* = 466 adults (57% females) referred for emotional, practical or legal victim support following crime or traffic accidents. PTSD status at 1 year post-trauma was based on CAPS-IV for internal and PCL-5 for external validation.

**Results:** During internal validation, all models achieved excellent accuracy (AUC/sensitivity/specificity > 0.90). During external validation, sufficient accuracy was only achieved for the sex-combined XGBoost model (AUC = 0.73, sensitivity = 0.69, specificity = 0.68), including 22 items of demographic and health characteristics, trauma characteristics, peri-traumatic distress or dissociation, post-traumatic cognitions, PTSD symptoms and social support.

**Conclusion:** We developed an accurate externally validated short prognostic screening instrument for PTSD based on self-report questions that is applicable to a broad population of recently trauma-exposed civilians. This novel instrument enables timely identification of individuals at risk for PTSD following trauma, and research into early targeted interventions to prevent long-term PTSD for civilians following trauma.

## HIGHLIGHTS

- We developed an accurate externally validated short screening instrument for PTSD risk 1 year post-trauma.
- It includes 22 self-report questions obtained within 2 months post-trauma and is applicable to a broad population of recently trauma-exposed civilians.
- This novel instrument enables timely identification of individuals at risk for PTSD following trauma.

## Abstract (es)

**Antecedentes**: La identificación oportuna de las personas con riesgo de desarrollar TEPT tras un trauma es crucial para brindar intervenciones preventivas específicas. Las técnicas de aprendizaje automático son prometedoras para la obtención de instrumentos de cribado pronóstico precisos. Sin embargo, aún no se dispone de instrumentos de cribado de pronóstico precisos y validados externamente para su aplicación generalizada en civiles expuestos a traumas. Además, se desconoce si la precisión de los instrumentos de cribado pronóstico podría mejorarse si se desarrollaran estratificando por sexo.

**Objetivo**: Nuestro objetivo fue desarrollar un instrumento de cribado pronóstico para el TEPT, validado externamente, basado en información auto-reportada obtenida en los dos meses posteriores al trauma en dos cohortes independientes de civiles recientemente expuestos a traumas, utilizando técnicas de aprendizaje automático que permitieran la extracción de un cuestionario breve. Examinamos si los modelos separados para hombres y mujeres mejoraban la precisión pronóstica en comparación con los modelos combinados por sexo.

**Métodos:** Se desarrollaron modelos de aprendizaje automático pronóstico (CART y XGBoost) en una cohorte longitudinal de *N* = 327 adultos (38 % mujeres) que requerían evaluación por lesiones graves (sospechadas) en un servicio de urgencias. La validación externa se realizó en otra cohorte longitudinal de *N* = 466 adultos (57 % mujeres) derivados para recibir apoyo emocional, práctico o legal a víctimas de delitos o accidentes de tráfico. El diagnóstico de TEPT al año del trauma se basó en la CAPS-IV para la validación interna y en la PCL-5 para la validación externa.

**Resultados**: Durante la validación interna, todos los modelos alcanzaron una precisión excelente (AUC/sensibilidad/especificidad > 0.90). Durante la validación externa, solo el modelo XGBoost combinado por sexo alcanzó una precisión suficiente (AUC = 0.73, sensibilidad = 0.69, especificidad = 0.68), incluyendo 22 ítems de características demográficas y de salud, características del trauma, malestar o disociación peritraumática, cogniciones postraumáticas, síntomas de TEPT y apoyo social.

**Conclusión:** Desarrollamos un instrumento de cribado pronóstico breve, preciso y validado externamente para el TEPT, basado en preguntas de autoinforme y aplicable a una amplia población de civiles expuestos recientemente a un trauma. Este novedoso instrumento permite la identificación oportuna de personas en riesgo de desarrollar TEPT tras un trauma, así como la investigación de intervenciones tempranas y específicas para prevenir el TEPT a largo plazo en civiles que han sufrido un trauma.

Most individuals experience at least one potentially traumatic event (PTE) in their lifetime (e.g. 81.5% lifetime PTE prevalence in the Netherlands, Hoeboer et al., 2025). For a considerable proportion of exposed individuals, a traumatic event results in developing posttraumatic stress disorder (PTSD; e.g. 11.1% lifetime PTSD prevalence in the Netherlands, Hoeboer et al., 2025). PTSD is characterized by intrusions, avoidance, negative mood and cognitions, and alterations in arousal and reactivity (APA, 2013). Moreover, PTSD has been associated with a high risk of co-morbid anxiety and depression symptoms, reduced well-being and quality of life, and increased health-care and work-related costs (e.g. Geraerds et al., 2019; Karchoud et al., 2024a, 2024b; Kessler, 2000). We also observed these adverse psychological, functional and economic outcomes in our recent long-term data, together with a 4.8% PTSD prevalence 12–15 following trauma exposure, further underscoring the considerable risk of long-term PTSD (Karchoud et al., 2024a, 2024b). As PTSD by definition can only develop after traumatic events, the initial period post-trauma presents an opportunity for preventive interventions to reduce (long-term) PTSD and its associated adverse outcomes (e.g. Karchoud et al., 2024a, 2024b; Kessler, 2000). There are different approaches to determine which individuals should receive preventive interventions (U.S. Institute of Medicine Committee on Prevention of Mental Disorders; see Mrazek & Haggerty, 1994). Increasing evidence suggests that preventive interventions for PTSD should not be provided to all trauma-exposed individuals (i.e. universal interventions), as it is more promising to target preventive interventions to those at high risk for developing PTSD (i.e. selective interventions) or those with substantial early PTSD symptoms (i.e. indicated interventions; e.g. Ennis et al., 2018; Garcia & Delahanty, 2017). For example, systematic reviews of preventive interventions for individuals exposed to traumatic events showed that targeted preventive interventions resulted in better improvements in mental health outcomes compared to preventive interventions delivered to all trauma-exposed individuals (Bisson et al., 2021; Ennis et al., 2018). In order to target preventive interventions towards individuals who need them most and are most likely to benefit, it is crucial to timely and accurately identify individuals at risk for developing PTSD following trauma.

Previous studies identified early post-trauma risk and protective factors for later development of PTSD across various domains, such as demographic, socio-economic, psychiatric, psychosocial, biological, trauma history and environmental factors (see e.g. Tortella-Feliu et al., 2019 for umbrella review of systematic reviews and meta-analyses on risk factors for PTSD). A large body of studies has examined such risk and protective factors using traditional statistical approaches investigating linear associations (Tortella-Feliu et al., 2019, e.g. van der Mei et al., 2020). Currently, machine learning methods are increasingly applied to predict PTSD based on these previously identified risk and protective factors (see systematic reviews and meta-analysis Blekic et al., 2025 and Vali et al., 2025). These machine learning studies demonstrate that these previously observed risk and protective factors are indeed relevant for making individual predictions (Blekic et al., 2025). Multiple studies across various civilian populations have shown good classification accuracy of internally validated prognostic models, meaning models were derived in a subset of their sample (i.e. training set) and accuracy was subsequently tested in a separate set of their sample (i.e. test set; Schultebraucks et al., 2021; Hinrichs et al., 2019; Galatzer-Levy et al., 2017; Papini et al., 2018). A recent meta-analytic study showed a pooled AUC (Area Under the Receiver Operator Curve) of 0.81 for these internal validation-based prognostic models (Vali et al., 2025), which supports machine learning as a promising computational method to derive prognostic screening instruments for PTSD. However, such internally validated models have a considerable risk of overfitting, meaning that the model may have learned patterns specific to the training data which do not generalize well to new data. External validation (i.e. testing in independent sample) is necessary to ensure that derived screening models are generalizable beyond their original samples and the resulting screening instrument will perform well upon implementation in practice (Altman et al., 2009; Vali et al., 2025). There are currently only a few studies that performed external validation of prognostic PTSD models, generally showing poor accuracy (pooled AUC = 0.59; Vali et al., 2025). This discrepancy in accuracy between internal and external validation illustrates that prognostic models can appear accurate during internal validation yet fail to perform adequate in new samples during external validation, thereby restricting their applicability in practice. This emphasizes the need for accurate externally validated models. A potential strategy to prevent overfitting to the training data during internal validation and achieve higher accuracy during external validation is to use less complex machine learning models (Zhang et al., 2022).

Successful implementation of a screening instrument in practice depends not only on its accuracy but also on its feasibility. The previously mentioned externally validated classification models mainly rely on acute biomedical assessments and information retrieved from hospital patients’ records, limiting their applicability beyond acute medical care settings (Schultebraucks et al., 2021; Hinrichs et al., 2019; Galatzer-Levy et al., 2017; Papini et al., 2018). Including only self-report items would likely promote large-scale applicability within a broader population, as this would allow recently trauma-exposed individuals to complete the assessment independently without the need for a healthcare professional. This approach could also increase uptake and acceptability of a screening instrument by promoting user empowerment, engagement, and a sense of self-control (Huygens et al., 2016). Previous machine learning studies based on self-reported data early post-trauma were able to predict subsequent PTSD with fair accuracy in internal validation sets (AUC > 0.70; Kim et al., 2023; Papini et al., 2023). However, data within these models were collected either already at the emergency department (ED) during the first 24 h post-trauma limiting the applicability beyond acute medical care settings (AUC = 0.79 Kim et al., 2023), or in highly specific populations such as military personnel limiting the generalizability to broader trauma-exposed populations (AUC = 0.74; Papini et al., 2023). None of the models based solely on self-reported data have been externally validated in recently trauma-exposed civilians (Vali et al., 2025). Thus, there is still a need for an accurate externally validated risk screening instrument for end-point PTSD status based on self-reported data that is both feasible to implement in practice and is widely applicable to a broad range of trauma-exposed civilians. This may include purposefully identifying a minimal number of risk and protective factors into prognostic screening models to ensure they remain practical and efficient for real-world use (Vali et al., 2025). Moreover, no study has yet examined whether stratifying prognostic models by sex and/or gender improves accuracy. Given the previously observed difference in PTSD prevalence and differential prognostic value of various risk and protective factors for PTSD between men and women (Haering et al., 2024a; 2024b; 2024c; Tolin & Foa, 2008), applying sex-stratified models may offer a more precise approach to PTSD risk assessment.

The aim of the current study was to develop a short, accurate externally validated prognostic PTSD risk screening instrument based on self-report information in recently trauma-exposed civilians. We included 180 prognostic variables based on previously found risk and protective factors for PTSD (see e.g. Tortella-Feliu et al., 2019 for umbrella review). Within this study we first derived prognostic models for end-point PTSD status at 1 year post-trauma in ED patients after (suspected) serious injury based on self-report information collected within the first 2 months post-trauma. Our goal was to find a balance between the complexity of machine learning models and the usability of a future screening instrument. Subsequently, we externally validated the derived prognostic models for end-point PTSD status in a sample of recent victims of traffic accidents and crime, using the same self-report questions, time periods since trauma for the prognostic information and the outcome, and algorithms. We developed prognostic models in females and males separately as well as a sex-combined model to examine whether sex-differential screening instruments may be relevant for improving early PTSD risk detection.

## 1. Methods

### 1.1. Participants and study design

#### 1.1.1 Model development sample

The model development sample was derived from the TraumaTIPS cohort (‘The Incidence, Prediction and Prevention of Post-trauma Psychopathology Study’; see Mouthaan et al., 2014 for more details). This sample consisted of *N* = 327 adults (37.6% female; mean age = 44.14 years, *SD* = 15.51) transported for medical evaluation of (suspected) serious injury by ambulance or helicopter to a level-1 emergency department (ED) of former hospitals in Amsterdam, the Netherlands (Academic Medical Center and VU University Medical Center, currently merged into Amsterdam University Medical Center) between 2005 and 2008. Participants were followed up to 1 year post-trauma. Inclusion criteria were: age 18 years or older; proficiency in Dutch; exposure to traumatic event according to Diagnostic and Statistical Manual of Mental Disorders 4th edition (DSM-IV) PTSD A1 criterion. Exclusion criteria were: current severe psychiatric symptoms (psychosis or schizophrenia; severe personality disorders; injuries resulting from deliberate self-harm); moderate-severe traumatic brain injury; permanent residency outside the Netherlands. The TraumaTIPS cohort study was approved by the Medical Ethics Review Committee of both hospitals (registration numbers 05-054# 05.17.0504; 06/039).

#### 1.1.2 External validation sample

The external validation sample was derived from the 2-ASAP cohort (‘Towards Accurate Screening and Prevention for PTSD; see Karchoud et al., 2024a, 2024b for more details). This sample consisted of *N* = 466 adults (57.4% female; mean age = 46.69 years, *SD* = 17.97) referred (mostly by the police) for emotional, practical or legal support following crime or traffic accidents to Victim Support Netherlands (Slachtofferhulp Nederland) between 2022 and 2023. Victim Support Netherlands is the largest non-profit organization in the Netherlands to provide nationwide emotional support as well as practical and legal support related to the criminal justice and damage compensation process after accidents, crimes or calamities. Victim Support Netherlands provides support in person, by telephone or online. While they help individuals recognize and manage stress-related complaints, they do not provide professional psychological treatment and instead refer victims to their general practitioner if indicated. Participants were followed up to 1 year post-trauma. Inclusion criteria were: age 18 years or older; experience of a traumatic event according to Diagnostic and Statistical Manual of Mental Disorders 5th edition (DSM-5) PTSD A criterion maximally 2 months post-trauma at baseline; in the form of direct exposure to events with an acute onset; external cause of a civilian nature; and the potential to lead to serious physical injury. Exclusion criteria were: evidence of homocidality or suicidality (i.e. having attempted to kill oneself or another person); injuries due to intentional self-inflicted injury; evidence of ongoing or repeated trauma exposure, such as ongoing domestic violence; evidence of an inability to understand study procedures, risks or being otherwise unable to give informed consent; evidence of being unable to reliably follow protocol (including visual or cognitive or physical impairment precluding completion of protocol); impairment in ability to use or no regular access to e-mail and internet-connected smartphone, tablet or computer; insufficient understanding of Dutch language to follow protocol. The 2-ASAP cohort study was approved by the Medical Ethics Review Committee of Amsterdam UMC (registration number 2022.0030).

The 2-ASAP cohort included more participants who were exposed to traumatic events involving physical assault, while the TraumaTIPS cohort included more occupational, domestic or recreational accidents (Pearson Chi-square = 160.05, *p* < .001). Participants of the 2-ASAP cohort were also less often injured as a result of their traumatic event compared to those in the TraumaTIPS cohort (Pearson Chi-square = 22.37, *p* < .001). Furthermore, the 2-ASAP cohort included more females (Pearson Chi-Square = 29.55, *p* < .001), more participants who were currently employed (Pearson Chi-Square = 134.95, *p* < .001), in a relationship without living together (Pearson Chi-Square = 10.45, *p* = .034), and with higher education levels (Pearson Chi-Square = 61.82, *p* < .001) compared to the TraumaTIPS cohort. There were no significant cohort differences in parental status (*p* = .606) and age (*p* = .50). See Table 1 for sample characteristics at baseline and end-point PTSD measurements of both the model development sample (TraumaTIPS Cohort: ED Amsterdam UMC) and external validation sample (2-ASAP Cohort: Victim Support Netherlands).

**Table 1.** Sample characteristics of model development sample (TraumaTIPS cohort: ED Amsterdam UMC) and external validation sample (2-ASAP cohort: Victim Support Netherlands) at baseline assessment < 2 months post-trauma.

	Model Development sample (TraumaTIPS cohort, *N *= 327) *n*	External Validation sample (2-ASAP cohort, *N *= 446) *n*
Sex (females)	123 (37.6%)	256 (57.4%)
Age in years at baseline, *M* (*SD*)	44.14 (15.51)	46.69 (17.97)
Relationship status at baseline		
Married/cohabitating/committed relationship	213 (65.1%)	324 (72.6%)
Divorced/widowed	27 (8.3%)	26 (5.8%)
No committed relationship	87 (26.6%)	96 (21.5%)
Children (yes) at baseline	184 (56.3%)	260 (58.3%)
Currently employed at baseline	73 (22.3%)	307 (68.8%)
Education, highest completed		
Primary education/high school/secondary education	143 (43.7%)	128 (28.8%)
Secondary vocational education	90 (27.5%)	106 (23.8%)
Higher vocational education or University	77 (23.6%)	205 (46%)
Trauma type		
Traffic accident	221 (67.6%)	298 (66.8%)
Physical assault	9 (2.7%)	131 (30.4%)
Occupational/domestic/recreational accident	93 (28.4%)	14 (3.8%)
Other (including e.g. natural disaster, fire or explosion)	4 (1.2%)	0
Injuries due to index trauma (yes)	312 (95.4%)	378 (84.8%)
PTSD symptom severity at 1 year post-trauma, *M* (*SD*)	16.01 (17.56)	12.48 (13.03)
End-point PTSD at 1 post-trauma (yes)	34 (8.3%)	48 (10.8%)

### 1.2. Procedures

#### 1.2.1 Model development sample

After medical evaluation in the ED, potential participants for the TraumaTIPS cohort were identified by screening hospital patient records regarding the inclusion and exclusion criteria. Further eligibility screening was performed in the hospital or via telephone within 72 h post-trauma. At the baseline assessment (T0), participants were screened for the exclusion criteria of current severe psychiatric symptoms using the Mini International Neuropsychiatric Interview (MINI; Plus version 5.0; Sheehan et al., 1998; Van Vliet & De Beurs, 2007), and provided written and oral informed consent. Participants completed self-report questionnaires on potential risk and protective factors for PTSD at baseline and the first follow-up within 2 months post-trauma (range = 1–60 days, *M* *=* 23.57*, SD* = 13.44). They received the questionnaire on paper. Participants were followed up to 1 year post-trauma with PTSD symptoms assessed via diagnostic interview using the Clinician-Administered PTSD scale for DSM-IV (CAPS-IV; Hovens et al., 1994; Weathers et al., 2004) at 3, 9 and 12 months. We used the CAPS-IV at 12 months post-trauma (range = 316–744 days, *M* = 427.33, *SD* = 69.32) as outcome variable for end-point PTSD status.

#### 1.2.2 External validation sample

Victim Support Netherlands identified potential participants for the 2-ASAP cohort via client records and sent them a letter via postal services, inviting them to contact the research team at Amsterdam UMC if they were interested in study participation. Potential participants who expressed interest in study participation, received additional study information and were called for eligibility screening (T0). Upon meeting all inclusion criteria and none of the exclusion criteria, informed consent was obtained through postal services. After inclusion, participants completed a baseline (T1) assessment within 2 months post-trauma (range = 16–60 days, *M* *=* 46.61*, SD* = 8.15), during which they completed the same self-report questionnaires on potential risk and protective factors for PTSD as in the model development sample. They received a personal link to the online questionnaire in Castor Electronic Data Capture (EDC). Participants were followed up to 1 year post-trauma with PTSD symptoms assessed using the PTSD checklist for DSM-5 (PCL-5; Boeschoten et al., 2014; Blevins et al., 2015; Hoeboer et al., 2024) at 3, 6, 9 and 12 months post-trauma. We used the PCL-5 at 12 months post-trauma (range = 360–434 days, *M* = 367.07, *SD* = 11.56) as outcome variable for end-point PTSD status.

### 1.3. Measures

#### 1.3.1. Outcome variable: end-point PTSD status

*CAPS-IV*. The Dutch version of the CAPS-IV was used to assess end-point PTSD status in the model development sample (Hovens et al., 1994; Weathers et al., 2004). The CAPS-IV consists of 17 items that correspond to DSM-IV PTSD symptom criteria (5 items for re-experiencing; 7 items for avoidance; 5 items for hyperarousal), assessing both frequency and intensity of each symptom in the past month on a 4-point Likert scale, ranging from 0 ‘absent’ to 4 ‘extremely’. PTSD symptom severity total scores were calculated by summing frequency and intensity scores for all 17 items (range 0-136, with higher scores reflecting higher symptom severity). The CAPS-IV has excellent internal consistency (Hovens et al., 1994; Weathers et al., 2004). We used a cut-off total score of 45 as indicative of a probable PTSD diagnosis (Weathers et al., 1999).

*PCL-5*. The Dutch version of the PCL-5 was used to assess end-point PTSD status in the external validation sample (Blevins et al., 2015; Boeschoten et al., 2014; Boeschoten et al., 2018). The PCL-5 consists of 20 items that corresponds to DSM-5 PTSD symptom criteria (5 items for intrusions; 2 items for avoidance; 7 items for negative alterations in cognitions and mood; 6 items for hyperarousal), assessing how much participants have been bothered by each symptom on a 5-point Likert scale ranging from 0 ‘not at all’ to 5 ‘extremely’ in the past month. PTSD symptom severity total scores were calculated by summing all item scores (range 0-80, with higher scores reflecting higher symptom severity; Chronbach’s α current sample = 0.94). We used a previously established cut-off score of 29, which was identified as most accurate threshold for estimating probable PTSD prevalence based on the CAPS-5 (Hoeboer et al., 2024). This validation study was conducted in a follow-up study of the current TraumaTIPS cohort and showed high convergence between self-reported (i.e. PCL-5) and clinical assessment of PTSD symptom severity total scores (i.e. CAPS-5; Hoeboer et al., 2024; see also Hoeboer et al., 2025).

#### 1.3.2 Prognostic variables: early-post trauma predictors

We evaluated 180 prognostic variables that were assessed early post-trauma (<2 months) from the following domains: demographic and health characteristics; medical and psychiatric history; psychological and physical health symptoms; current trauma characteristics and related acute emotions, posttraumatic cognitions and symptoms; perceived social support; prior trauma exposure. See Supplementary file A for an overview of all included prognostic variables.

#### 1.3.3 Sex

Within the TraumaTIPS cohort sex assigned at birth (i.e. 62.4% male, 37.6% female) was retrieved from hospital records. Within the 2-ASAP cohort we assessed both sex assigned at birth and gender identity. We asked participants their sex assigned at birth (i.e. 42.6% male, 57.4% female), and whether they self-identified as men (43%), women (56.7%) or otherwise (0.2%). These percentages indicate substantial overlap between sex assigned at birth and gender identity within this sample. We chose to focus on sex assigned at birth for consistency with the TraumaTIPS cohort.

### 1.4. Statistical analyses

The statistical analyses plan was pre-registered on OSF (Karchoud et al., 2024a, 2024b). Statistical analyses were performed using R Version 3.6.1 and IBM SPSS Statistics Version 28.0. We reported all information necessary for quality assessment (i.e. risk of bias and applicability) of our prediction models using the ‘Prediction model Risk of Bias ASsement Tool’ (PROBAST) guidelines (Moons et al., 2019).

#### 1.4.1 Missing data imputation

See Supplementary file A for an overview of missing data in the model development and external validations samples.

*Model Development Sample*. The percentage of missing data for prognostic variables in the model development sample ranged from 0% to 29.7%. Missing data was imputed prior to splitting the data into test/training sets (Tang & Ishwaran, 2017), using the non-parametric Random Forest algorithm to impute both categorical and continuous predictor data (Stekhoven & Bühlmann, 2012). We used the R package ‘missForest’ with 5 iterations (Stekhoven & Bühlmann, 2012). While Stekhoven and Bühlmann (2012) calculated Normalized Root-Mean-Squared Error (NRMSE) to evaluate performance of imputation, we chose not to standardize our variables given that variable distributions may differ between the model development and external validation samples. Performance of imputation was good based on the proportion of Falsely Classified (PFC = 0.12) and Root-Mean-Squared Error (RMSE = 0.63, higher scores due to non-standardized data; Stekhoven & Bühlmann, 2012).

*External Validation Sample*. The percentage of missing data of predictors variables in the external validation sample ranged from 0% to 5.8%, except for one variable measuring working hours per week with a missingness of 51.8%, because many participants who worked did not specify the follow-up question of amount of working hours per week. Missing data was imputed the same way as in the model development sample, using the R package ‘missForest’ with 4 iterations (Stekhoven & Bühlmann, 2012). Given that the external validation sample contained limited missing data (see Supplementary File A for an overview), the imputation procedure is unlikely to have influenced the external validation results. Performance of imputation was good based on the proportion of Falsely Classified (PFC = 0.01) and Root-Mean-Squared Error (RMSE = 0.92, higher scores due to non-standardized data; Stekhoven & Bühlmann, 2012).

#### 1.4.2 Development of prognostic machine learning models

We developed prognostic machine learning models to predict end-point PTSD status, separately for males and females, and for both sexes combined. We conducted random synthetic oversampling of the minority class (i.e. those with PTSD) in males and females separately to ensure balanced classes of the outcome variables, as recommended to deal with imbalanced classes (López et al., 2013). For the sex combined sample, we applied oversampling within each sex separately to ensure an equal proportion of males and females (50%), in order to avoid biased model estimations for the underrepresented sex (Langeland & Olff, 2024). We used the ‘imbalance’ package in R for Majority Weighted Minority Oversampling Technique (MWMOTE; extension of synthetic minority over-sampling technique (SMOTE) algorithm; Cordón et al., 2018). The samples were randomly split into a 65% training set for model building and 35% test set for internal validation of the derived model.

We followed a stepwise approach of conducting different machine learning models to achieve good classification accuracy for internal and external validation. For internal validation, we aimed to achieve good accuracy (AUC = 0.80, sensitivity = 0.75, specificity = 0.70) as the minimally acceptable accuracy for our prognostic models. For external validation, we aimed to achieve fair accuracy (AUC = 0.70) and a sensitivity of 0.65 and specificity of 0.60, considering that external validation generally results in lower accuracy (Siontis et al., 2015; Vali et al., 2025). We choose to perform tree-based models, given results of a meta-analytic study showing that tree-based models generally performed better in predicting PTSD than other models (Vali et al., 2025). We evaluated 180 potential risk and protective factors as input variables in the algorithms, from which the algorithm derived a minimal feature set consisting of the variables included in the final models. First, we performed the Classification And Regression Tree (CART; Breiman et al., 2017), a pragmatic tree-based algorithm suited for our goal to identify a minimal set of variables in the prognostic models. We performed CART using the ‘rpart’ package in R (Therneau et al., 2015). We build an initial tree using 5-fold cross-validation on the training set, while avoiding excessive complexity of the model to avoid the risk of overfitting (i.e. maximum depth of tree = 10; complexity parameter = 0.005; minimum number of observations required to split a node = 5). Subsequently, we used a pruning strategy to optimize the amount of features within the models by determining the complexity parameter based on the ‘1-SE’ rule (Breiman et al., 2017). Second, we proceeded with a more advanced tree-based algorithm, Extreme Gradient Boosting (XGBoost; Chen et al., 2015), an ensemble tree-based method that combines multiple decision trees to improve classification accuracy through iterative boosting. We performed XGBoost using the ‘caret’ and ‘xgboost’ packages in R (Chen et al., 2019; Kuhn, 2008). We build a tree using 5-fold cross-validation on the training set, while avoiding excessive complexity of the model to avoid the risk of overfitting and to identify a minimal feature set (i.e. number of trees = 50; maximum depth of tree = 1; eta learning rate = 0.2; gamma complexity regularization = 3; subsample size per iteration = 0.7; percentages features per tree = 0.5; minimal child weight for tree node = 2). The final models generated from the training sets were applied to the test sets to calculate the accuracy parameters. We calculated the overall accuracy (i.e. ratio correct predictions and all predictions); AUC (Area Under the Receiver Operator Curve; i.e. capability to distinguish different classes); sensitivity (i.e. proportion true positives correctly classified); specificity (i.e. proportion true negatives correctly classified); precision (i.e. ratio true positives and all predictions); recall (i.e. ratio true positives and actual positive predictions).

#### 1.4.3 External validation of derived models

External validation was performed on the derived models of end-point PTSD status for females and males separately and in a sex-combined sample. The algorithms generated from the model development sample were applied to the external validation test set to calculate the same accuracy parameters as for internal validation (i.e. overall accuracy; AUC; sensitivity; specificity; precision; recall).

#### 1.4.4 SHapley Additive exPlanations (SHAP)

To provide interpretations of our derived machine learning model, we applied SHapley Additive exPlanations (SHAP), using the ‘shapviz‘ and ‘kernelshap’ packages in R (Lundberg & Lee, 2017). SHAP is used for decision tree-based non-linear models, such as XGBoost, to allow for interpretable insights into how individual variables contribute to the model’s prediction (Lundberg & Lee, 2017). We assessed the relative importance of each predictor in the final model (i.e. SHAP values), and the direction of its influence on the predicted outcome (i.e. feature values).

## 2. Results

### 2.1. Model development and validation

See Table 2 for an overview of the accuracy parameters for internal and external validation of CART and XGBoost prognostic models for end-point PTSD status 1 year post-trauma in males, females, and sex combined.

**Table 2.** Accuracy of CART and XGBoost prognostic models for end-point PTSD status in males, females, and sex combined.

	*n* variables in model	Internal validation Accuracy (95% CI)	Internal validation AUC; Sensitivity; Specificity	Internal validation Precision; Recall	External validation Accuracy (95% CI)	External validation AUC; Sensitivity; Specificity	External validation Precision; Recall
**CART model**	* *						
Females	4	95% (0.88–0.99)	0.95; 0.98; 0.93	0.93; 0.98	80% (0.75–0.85)	0.45; 0.00; 0.90	0.00; 0.00
Males	4	97% (0.93–0.99)	0.97; 1.00; 0.94	0.94; 1.00	86% (0.81–0.91)	0.55; 0.16; 0.94	0.23; 0.16
Sex combined	6	94% (0.89–0.97)	0.96; 0.96; 0.93	0.85; 0.96	81% (0.77–0.84)	0.68; 0.31; 0.87	0.22; 0.31
**XGBoost model**							
Females	19	95% (0.88–0.99)	0.98; 0.98 0.93	0.93; 0.98	82% (0.76–0.86)	0.57; 0.03; 0.92	0.05; 0.03
Males	19	96% (0.92–0.99)	1.00; 1.00; 0.93	0.93; 1.00	80% (0.74–0.85)	0.74; 0.53; 0.83	0.26; 0.53
Sex combined	22	93% (0.89–0.94)	0.98; 0.92; 0.93	0.86; 0.89	68% (0.63–0.72)	0.73; 0.69; 0.68	0.20; 0.69

#### 2.1.1 Internal validation

We achieved excellent accuracy for all sex-specific and sex-combined models in the internal validation test sets, using CART (overall accuracy = 94-97%, AUC = 0.95–0.97, sensitivity 0.96–1.00, specificity 0.93–0.94) and XGBoost (overall accuracy = 93-96%, AUC = 0.98–1.00, sensitivity 0.92–1.00, specificity 0.93).

#### 2.1.2 External validation

External validation of the CART sex-combined model resulted in poor accuracy (AUC = 0.68; specificity = 0.87; sensitivity = 0.16). This model performed better than the separate models for males (AUC = 0.55, sensitivity = 0.16, specificity = 0.94) and females (AUC = 0.45, sensitivity = 0.00, specificity = 0.90).

External validation of the XGBoost sex-combined model resulted in fair accuracy (AUC = 0.73) with a specificity of 0.69 and sensitivity of 0.68. This model performed better than the separate models for males with fair accuracy (AUC = 0.74) but a poor sensitivity of 0.53 and a specificity of 0.83; and females with poor accuracy (AUC = 0.57, sensitivity = 0.03, specificity = 0.92).

### 2.2. Prognostic screener for end-point PTSD status

**Figure 1.**
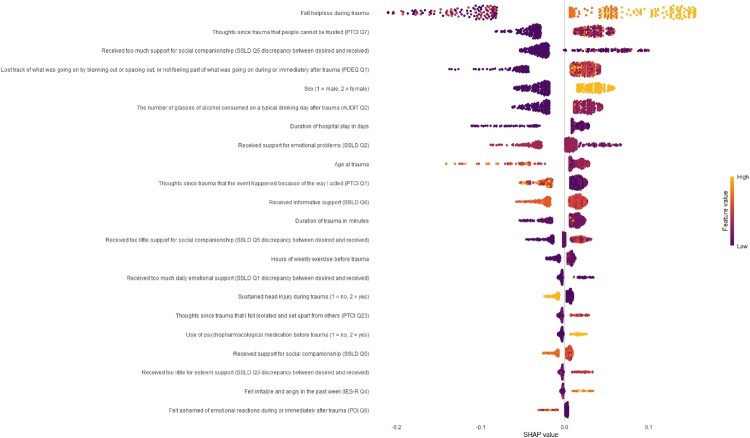
SHAP values indicating the relative importance of each predictor in the final derived sex-combined XGBoost model and the direction of its influence on end-point PTSD risk. Note. SHAP values indicating the relative importance of each predictor in the final derived model (i.e. higher absolute values on x-axis indicating greater importance; predictors on y-axis ranked from most to least important), and the direction of its influence (i.e. low to high feature values indicated by colour per participant) on the chance of developing end-point PTSD (i.e. positive SHAP values on x-axis) and of developing no end-point PTSD (i.e. negative SHAP values on x-axis).

The sex-combined XGBoost model was the only prognostic model that achieved sufficient external validation accuracy and was thus used to obtain the prognostic screening instrument for end-point PTSD status. The model included 22 questions from the following domains: demographic and health characteristics; current trauma characteristics; peri-traumatic distress or dissociation; post-traumatic cognitions; PTSD symptoms; social support. See Table 3 for an overview of each predictor per domain included in the final derived prognostic screener for end-point PTSD status. See Figure 1 for SHAP values indicating the relative importance of each predictor in the final derived model and the direction of its influence on the chance of developing end-point PTSD and of developing no end-point PTSD. See supplementary file B for instructions to use the algorithm that was used to derive the model.

**Table 3.** Overview of variables per domain included in the final prognostic screener for end-point PTSD status (sex-combined XGBoost model).

Included variables in prognostic screener for end-point PTSD status per domain
**Demographics and health characteristics** Sex; Age at trauma; Hours of weekly exercise before trauma; Use of psychopharmacological medication before trauma; The number of glasses of alcohol consumed on a typical drinking day in the past year (AUDIT Q2);
**Current trauma characteristics** Duration of hospital stay in days; Duration of trauma in minutes; Sustained head injury during trauma;
**Peri-traumatic distress or dissociation** Lost track of what was going on by blanking out or spacing out, or not feeling part of what was going on during or immediately after trauma (PDEQ Q1); Felt ashamed of emotional reactions during or immediately after trauma (PDI Q6); Felt helpless during trauma;
**Post-traumatic cognitions** Thoughts since trauma that the event happened because of the way I acted (PTCI Q1); Thoughts since trauma that people cannot be trusted (PTCI Q7); Thoughts since trauma that I felt isolated and set apart from others (PTCI Q23);
**PTSD symptoms** Felt irritable and angry in the past week (IES-R Q4)
**Social support** Received support for emotional problems (SSLD Q2); Received informative support (SSLD Q6); Received support for social companionship (SSLD Q5); Received too little support for social companionship (SSLD Q5 discrepancy between desired and received); Received too little for esteem support (SSLD Q3 discrepancy between desired and received); Received too much support for social companionship (SSLD Q5 discrepancy between desired and received); Received too much daily emotional support (SSLD Q1 discrepancy between desired and received).

Note*:* AUDIT: Alcohol Use Disorders Identification Test (Bush et al., 1998); PDEQ: Peritraumatic Dissociative Experiences Questionnaire (Marmar et al., 2004); PDI: Peritraumatic Distress Inventory (Brunet et al., 2001); PTCI: Posttramatic Cognitions Inventory (Foa et al., 1999); IES-R: Impact of Event Scale Revised (Weiss, 2007); VVV: Verkorte Vermoeidsheidsvragenlijst (Alberts et al., 1997); HADS: Hospital Anxiety and Depression Scale (Spinhoven et al., 1997); SSLD: Sociale Steun Lijst Discrepanties (van Sonderen, 1997).

## 3. Discussion

The aim of the study was to develop an externally validated prognostic screening instrument for PTSD that is applicable to recently trauma-exposed civilians. To achieve this, we used machine learning techniques to extract a short prognostic screening instrument for end-point PTSD status 1 year post-trauma based on self-report information obtained within 2 months post-trauma. The prognostic models were first developed in an emergency department cohort primarily exposed to traffic accidents and occupational, domestic or recreational accidents involving (suspected) serious injury. Subsequently, we performed external validation on the derived models to test the generalizability in a cohort from a national victim support organization with more heterogeneous types of trauma (i.e. more physical assault and also traffic accidents), and who were less likely to be injured, and less severely injured. In addition to recruitment centre and trauma type, the cohorts also differed in socio-demographic characteristics, including sex, education level, relationship status, and current employment status. We also compared the accuracy of separate models for males and females and sex-combined models to examine whether sex-differential screening instruments may be relevant for improving prognostic screening performance. The only model with fair accuracy for external validation was the sex-combined XGBoost model (AUC = 0.73; specificity = 0.69; sensitivity = 0.68). The differences between cohorts further underscores our model’s likely generalizability to different populations of recently trauma-exposed civilians, thereby highlighting its potential for real-world implementation.

Within our study we included 180 predictors (i.e. self-report items) in the algorithm based on previously established risk and protective factors for PTSD (see systematic review Tortella-Feliu et al., 2019). Our final derived screening instrument included 22 self-report items. The most important predictor for PTSD risk was feeling helpless during trauma. We found multiple predictors related to demographic, health and trauma characteristics, which were all already well-documented risk factors for PTSD, particularly females and younger individuals are often considered at higher risk for PTSD, as well as trauma severity indicators such as duration of hospital stay (Tolin & Foa, 2008; Tortella-Feliu et al., 2019). Additionally, the same or similar predictors as in our model have also been reported in a recent systematic review of longitudinal machine learning studies on predictors of PTSD (i.e. sex; younger age; previous psychological treatment; peri-traumatic dissociation and distress; post-traumatic cognitions; PTSD symptoms; alcohol use and perceived social support; Blekic et al., 2025). Notably, some of these predictors were also supported in external validation studies (i.e. younger age; psychiatric history; alcohol use; peri-traumatic distress and dissociation; and social support; Blekic et al., 2025). Within these studies external validation was performed in a similar sample in which the model was built, both ED cohorts (Schultebraucks et al., 2020) or comparable military deployment cohorts (Karstoft et al., 2020; Papini et al., 2023). Our study, including external validation in a different cohort type, extends these findings by demonstrating that similar predictors hold in different populations of recently trauma-exposed civilians.

A recent systematic review of machine learning studies examined whether predictors identified using these data-driven methods align with leading theoretical models of PTSD (Blekic et al., 2025). They reported multiple predictors that we also found within this study regarding psychiatric history; peri-traumatic distress and dissociation; as well as social support; that all align with the cognitive model of PTSD (Ehlers & Clark, 2000). Our model additionally identified predictors of post-traumatic cognitions (i.e. mistrust in others, self-blame and feelings of isolation) that align with both the cognitive model (Ehlers & Clark, 2000) as well as the social cognitive model (Sharp et al., 2012), emphasizing maladaptive understandings of the self- and other that hinder social support and increase the vulnerability of developing PTSD. This is also consistent with our derived predictors related to discrepancies in received and desired social support, with both too little and too much perceived social support as risk factor for PTSD. This social support domain was most represented in the final model. On the one hand, this is not surprising as there is robust evidence for the importance of social support as protective factor for trauma-related disorders (Brewin et al., 2000; Maercker, 2025; Santos et al., 2025; Wang et al., 2021). On the other hand, it may be considered surprising that the final model only included one PTSD symptom as relevant (i.e. felt irritable and angry). Our results suggest that early risk screening for PTSD based on risk and protective factors across multiple domains may be more accurate, rather than relying solely on acute PTSD symptoms alone as captured by validated PTSD symptom screeners such as the Impact Event Scale Revised (IES-R; Christianson & Marren, 2012). Future research should directly compare the accuracy of our derived multi-domain data-driven prognostic screening instrument based on machine learning algorithms to the accuracy of such validated PTSD symptom screeners that assess current PTSD, screening instrument based on theoretical constructs, and other best practice screening instruments that are currently available for PTSD, such as the primary care PTSD screen for DSM-5 (PC-PTSD-5; Prins et al., 2016); Global Psychotrauma Screen (GPS; Frewen et al., 2021; Olff et al., 2020). Whilst our application of SHAP provided a first step into understanding the relative contributions of the 22 items within the derived algorithm, future research could also apply network analyses to investigate whether identified risk and protective factors are similary associated amongst individuals with and without high risk for PTSD. Taken together, our findings demonstrate considerable convergence in predictors with previous machine learning studies and theoretical models for PTSD, while also extending the literature by highlighting the utility of self-report questions in a broad population of recently trauma-exposed civilians.

Within this study we tried to find a balance between model complexity and practicality for use of a screening instrument with fewer items. We started with a relatively simple tree-based algorithm (i.e. CART), however this did not generalize well as we found poor accuracy during external validation. Subsequently we performed an ensemble method that combines multiple decision trees which improves generalization to unseen cases (i.e. XGBoost algorithm; Chen et al., 2015). The sex-combined XGBoost model achieved higher accuracy for external validation (AUC = 0.73) than the pooled prevalence of the few studies that performed external validation of prognostic PTSD models thus far (pooled AUCs = 0.59; Vali et al., 2025). The poorer accuracy of external validation observed in previous studies has been proposed to result from overfitting during internal validation due to the use of complex machine learning models that are overfitted to the training set (Zhang et al., 2022). Although other studies also performed XGBoost, we simplified the model by adjusting complexity parameters during model development to limit the number of items selected by the machine learning model (e.g. maximum depth of a tree). We did not build more complex models (e.g. allowing deeper trees), as this would have conflicted with the study aim to develop a concise screening instrument. Moreover, this also prevented the model from overfitting to the training set. Although our derived model achieved fair prognostic accuracy for external validation (AUC = 0.73), the model achieved modest sensitivity (0.69), specificity (0.68), recall (0.69) and precision (0.20). This means that while the model correctly identified the majority of individuals who develop PTSD (i.e. true positives), it also incorrectly classified a substantial number of individuals at risk who ultimately do not develop PTSD (i.e. false positives). While this low precision is not ideal, it is not necessarily problematic in a screening context, as these individuals could still experience subclinical PTSD symptoms or other adverse psychological outcomes and as such may still benefit from preventive interventions. Additionally, the clinical relevance of subclinical PTSD and its potential to cause distress and impairment is now acknowledged by the DSM-5 (i.e. under ‘Other Specified Trauma- and Stressor-Related Disorder’). Thus, even with limited precision, the model may serve a valuable role in identifying those who are most likely to benefit from preventive intervention.

Future research should keep improving classification accuracy by further updating these prognostic machine learning models. We built our prognostic models based on previously established risk and protective factors that were established at the time of data collection in the model development sample (TraumaTPS cohort; Mouthaan et al., 2014), but it may also be valuable to explore other potential risk and protective factors for PTSD, for example those identified in the systematic review of machine learning studies with external validation sets (Blekic et al., 2025). Within this study we used self-report questions to promote large-scale applicability of the derived screening instrument for PTSD risk. In particular, it would be interesting to examine other predictors assessed via self-report questions, such as those related to sleep problems or coping strategies (Blekic et al., 2025).

Given that sex assigned at birth was found as predictor in our final model, future research could benefit from including sex-specific and gender-specific risk factors in stratified models, for example by examining factors related to ovarian steroid hormonal variation across the female lifespan (Wiseman et al., 2023) or related to types of interpersonal trauma more often experienced by women such as sexual assault (Hoeboer et al., 2025). Sex-specific models performed well for internal validation, but poor in the external validation phase, particularly for females compared to males. This could be explained overfitting due to the reduced sample size following stratification by sex (e.g. Larracy et al., 2021). We encourage other researchers to examine adequately powered sex-stratified machine learning models including sex- and gender-specific risk factors alongside sex-combined models. Besides improving prognostic accuracy, accurate sex-stratified models may also offer valuable insights into potential sex-specific risk and protective factors (see systematic review and meta-analysis on sex/gender differences in PTSD risk factors; Haering et al., 2024a, 2024b, 2024c).

The current study has several limitations. Our initial aim was to predict distinct courses of PTSD symptoms over time using latent trajectory analyses rather than a single end-point status of PTSD (see protocol study Karchoud et al., 2024a, 2024b). However, this turned out not to be feasible due to substantial differences in emerging latent PTSD symptom trajectories and model fit between the internal and external validation samples, which would have resulted in predicting fundamentally different outcomes across cohorts. In order to be able to perform external validation, we therefore focused on predicting end-point PTSD status instead, which still served the aim of the study to identify individuals at risk for developing PTSD over time. However, the potential impact of the use of different instruments to assess end-point PTSD status in the internal and external validation cohorts must be noted. Although previous research demonstrated high convergence between self-reported (i.e. PCL-5) and clinical assessment (i.e. CAPS-5) of PTSD symptom severity total scores (Hoeboer et al., 2024), the use of different outcome measures may have introduced variability in the classification of PTSD cases. Given that the model has been trained to capture PTSD based on the CAPS-IV assessment, the model may not fully capture PTSD based on the PCL-5 assessment. This may have affected the model’s accuracy during external validation, potentially missing individuals with PTSD based on the PCL-5 or falsely classifying individuals with PTSD based on the CAPS-IV. However, even if both cohorts had used clinician-rated assessments (i.e. CAPS), this would still have resulted in a discrepancy, as the internal cohort was assessed using the CAPS-IV based on DSM-IV criteria, while the external cohort would require the CAPS-5 based on DSM-5. Moreover, differences in the timing of data collection of the risk and protective factors for PTSD may have influenced model performance during external validation. Although both assessed within 2 months post-trauma, the predictors in the model development sample were assessed immediately post-trauma while this was a few weeks later in the external validation cohort. Potentially, the differences in the timing of the data collection of the included risk and protective factors in the model development and external validation samples may have influenced the PTSD symptom presentation, as the external validation sample was assessed at a later time point post-trauma and participants may therefore have been in a different phase of symptom progression or recovery. While these discrepancies between the model development and external validation cohorts may have introduced lower prediction accuracy, they also underscore the robustness of the derived model across diverse assessment contexts. Last, while the hospital records used for our model development and internal validation cohort most likely contained information on biological sex, it is not entirely certain whether sex or gender was assessed.

For future implementation purposes, we tested our derived prognostic models in another independent cohort of recently trauma-exposed civilians. We also focused on deriving short and interpretable models from large-scale self-report data. This minimizes overwhelming users with excessive item burden when filling in the screening instrument, supporting its potential use as a general prognostic screener for widespread implementation in real-world settings. This facilitates identification of individuals at risk for developing end-point PTSD 1 year post-trauma, enabling targeted preventive interventions for PTSD. The screener including the algorithm developed in this study will be freely available. We will pilot the clinical utility in a forthcoming randomized controlled trial (RCT) indicated early intervention for PTSD towards those recognized to be at high PTSD risk using the screener.

## 4. Conclusion

We developed a short screening instrument for PTSD risk in recently trauma-exposed civilians based on a machine learning algorithm using information derived from self-report questions. By externally validating the derived prognostic machine learning model, we demonstrated its generalizability across different populations of recently trauma-exposed civilians. This strengthens the potential for real-world implementation of our screening instrument for early PTSD risk screening. With this study, we take a step towards targeted early interventions for civilians in the aftermath of a traumatic event.

## Supplementary Material

Supplementary File B algoritm instructions anonymous ..docx

Supplementary File A Missing data Revision anon.docx

## Data Availability

The data of the study and the code to produce the results described in this paper are available at Open Science Framework (OSF; https://osf.io/59eax/). The 2-ASAP and TraumaTIPS cohorts are registered in the FAIR Traumatic Stress Data Sets library of the Global Collaboration on Traumatic Stress (GCTS).
