# Supplementary material for: Development and external validation of a short prognostic screening instrument for PTSD one year following individual civilian trauma
Source: Eur J Psychotraumatol. 2025 Dec 15;16(1):2594266. doi: 10.1080/20008066.2025.2594266 (PMC12707094; doi:10.1080/20008066.2025.2594266)
Supplement: Supplementary File B algoritm instructions anonymous ..docx [file ZEPT_A_2594266_SM1752.docx]

**Instructions for using the Screening instrument for PTSD risk 1 year post-trauma**

This document explains how to load and apply our derived prognostic model TT_xgb_both to your own data in R. Our analyses were performed using R version 3.6.1.

**Prepare your dataset**

Our dataset and data dictionary is available on Open Science Framework (OSF) via [anonymous]. Ensure your dataset contains the same features as used in our prognostic model and that the outcome variable Class is included. The features need identical variable names, values and categorization (i.e., factor or numeric) as within our dataset.

**Required R packages**

Xgboost (Chen et al., 2019); caret (Kuhn, 2008).

Chen, T., He, T., Benesty, M., & Khotilovich, V. (2019). Package ‘xgboost’. *R version*, *90*(1 66), 40.

Kuhn, M. (2008). Caret package. *Journal of statistical software*, *28*(5), 1-26.

**Model file**The model file TT_xgb_both.RData is available on OSF via [anonymous]. Download the file and place it in your working directory.

**Usage**

The R script to load the model and apply it to your own data is available on OSF via [anonymous].
