# Supplementary material for: Development and external validation of a short prognostic screening instrument for PTSD one year following individual civilian trauma
Source: Eur J Psychotraumatol. 2025 Dec 15;16(1):2594266. doi: 10.1080/20008066.2025.2594266 (PMC12707094; doi:10.1080/20008066.2025.2594266)
Supplement: Supplementary File A Missing data Revision anon.docx [file ZEPT_A_2594266_SM1750.docx]

**Supplementary File A**

| **Domain** | **Predictor variables** |
| --- | --- |
| Demographic and Health Characteristics | Time post-trauma; Sex; Age (in years); Highest completed education; Marital status; Children; Number of children; Children living at home; Employment or daytime activity; Hours per week working; Currently employed; Full-time / part-time employment; Chronic condition; Previous psychological complaints/disorders; Current medication use; Current treatment; Psychiatrist involved in treatment; Psychologist involved in treatment; Social worker involved in treatment; Hospital admission as part of treatment; Currently experiencing psychological symptoms/disorders; Family history of psychological disorders; Sports participation before admission; Hours per week (sports before admission); Sports participation at T2; Hours per week (sports at T2); Physical health/fatigue (feel tired, get tired easily, feel fit, feel physically exhausted); Frequency of drinking alcoholic beverages; Typical number of drinks per drinking day; Frequency of consuming 6+ drinks per occasion; Frequency unable to stop drinking once started; Frequency of neglecting responsibilities due to drinking; Frequency of morning drinking after heavy use; Frequency of guilt or remorse about drinking; Frequency of memory loss after drinking; Self or others injured due to drinking; Concern expressed by others about drinking |
| Current Trauma Characteristics | Categorization of traumatic event(s); Complete memory of the event; Amount remembered of the event; Time of trauma (in hours); Duration of trauma (in minutes); Thought they would die; Sustained injury; Injured: head; Injured: face; Injured: neck; Injured: lungs/heart/chest; Injured: thorax; Injured: abdomen; Injured: pelvis; Injured: back; Injured: upper limbs; Injured: lower limbs; Someone else injured; Someone died; Witnessed injured persons; Witnessed dead persons; Acquaintance injured; Acquaintance deceased; Hospital admission due to trauma; ICU admission; Length of hospital stay; Discharge location |
|  |  |
| Peri-Traumatic Distress or Dissociation | Afraid; Angry; Guilty; Powerless; Disgusted; Sad; Felt loss of control/confusion/blackout; Acting on “automatic pilot”; Altered sense of time; Sense of unreality; Feeling detached or watching from above; Body felt unreal/detached/distorted; Felt as if events happening to others were happening to oneself; Surprised by events that occurred without awareness; Confused about what was happening; Disoriented regarding time or place; Felt helpless to act; Felt frustrated or angry about inability to act; Worried about own safety; Felt guilty for not doing more; Felt ashamed of emotional reactions; Worried about safety of others; Felt unable to control emotions; Difficulty controlling bladder/bowels; Physically shocked by what was seen; Physical reactions (sweating, trembling, palpitations); Thought they might faint; Thought they might die |
|  |  |
| Post-Traumatic Cognitions | The event happened because of my actions; People cannot be trusted; Another person would not have ended up in this situation; I cannot rely on other people; I have no future; People are not what they seem; Something about me caused the event; I no longer know myself; Nothing good can happen to me anymore; The event happened due to the way I acted; I feel like I don’t know myself anymore; I feel powerless; I feel unsafe; I feel responsible for what happened |
| PTSD Symptoms | \| Feelings return when reminded of event; Difficulty staying asleep; Things reminded me repeatedly of the event; Irritable and angry; Tried not to get upset when reminded; Intrusive thoughts about the event; Felt as if it had not really happened; Avoided reminders of the event; Intrusive images of the event; Felt tense and easily startled; Avoided thinking about the event; Aware it still affected me but did nothing about it; Felt emotionally numb; Reexperienced the event as if reliving it; Difficulty falling asleep; Intense emotions at times; Tried to block it from memory; Difficulty concentrating; Physical reactions when reminded (sweating, nausea, etc.); Dreamed about the event; Hypervigilant / on guard; Avoided talking about it \| \| --- \| |
| Social Support | \| \| Everyday emotional support; Emotional support in problem situations; Appreciation; Instrumental support; Social companionship; Informational support; Lack of everyday emotional support; Lack of emotional support in problems; Lack of appreciation; Lack of instrumental support; Lack of social companionship; Lack of informational support; Excessive everyday emotional support; Excessive emotional support in problems; Excessive appreciation; Excessive instrumental support; Excessive social companionship; Excessive informational support \| \| --- \| \| \| --- \| --- \| |
|  |  |

**Table A**

*Missing data TraumaTIPS cohort (development sample) and 2-ASAP cohort (external validation sample)*

| TraumaTIPS cohort |  |  |
| --- | --- | --- |
| Lichamelijk voel ik me uitgeput | | 23,9% |
| Ik voel me fit | | 23,9% |
| Ik ben gauw moe | | 23,5% |
| Ik voel me moe | | 23,5% |
| hoelang duurde trauma in minuten | | 23,5% |
| Aantal uren per week | | 23,2% |
| De gebeurtenis vond plaats door de manier waarop ik handelde | | 22,3% |
| In mijn hoofd kwamen beelden van de gebeurtenis op | | 22,3% |
| Ik voelde me gefrustreerd of boos dat ik niet meer kon doen | | 22,3% |
| Ik voelde droevigheid en verdriet | | 22,3% |
| Mensen zijn niet wat ze lijken | | 22,0% |
| Ik merkte dat ik me weer zo gedroeg of me weer zo voelde alsof ik alles weer meemaakte | | 22,0% |
| Ik zorgde ervoor niet van streek te raken, wanneer ik eraan dacht of eraan herinnerd werd | | 22,0% |
| Ik was prikkelbaar en boos | | 22,0% |
| Ik voelde me schuldig dat er niet meer werd gedaan | | 22,0% |
| Ik was achteraf verbaasd dat een heleboel dingen waren gebeurd zonder dat ik me er bewust van was, vooral dingen die mij normaal opgevallen zouden zijn. | | 22,0% |
| Ik had het gevoel dat dingen die in feite anderen overkwamen nu mij overkwamen - alsof ik in de val was gelopen zonder dat dat werkelijk zo was | | 22,0% |
| Mij kan niets goed meer overkomen | | 21,7% |
| Het voelt alsof ik mezelf niet meer ken | | 21,7% |
| Ik was waakzaam of op mijn hoede | | 21,7% |
| Ik was me ervan bewust dat het me nog erg raakte, maaar ik deed er niets mee | | 21,7% |
| Zonder dat ik het wiilde, moest ik eraan denken | | 21,7% |
| Verschillende dingen deden mij er steeds weer aan denken | | 21,7% |
| Ik voelde me hulpeloos om meer te kunnen doen | | 21,7% |
| Ik had het gevoel dat ik als een toeschouwer keek naar wat er met mij gebeurde, alsof ik erboven zweefde of zat toe te kijken als een buitenstaander | | 21,7% |
| Wat er gebeurde leek onwerkelijk, alsof ik droomde of naar een film of toneelstuk zat te kijken | | 21,7% |
| Mijn tijdsgevoel veranderde - tijd leek traag te gaan | | 21,7% |
| Ik deed dingen op de "automatische piloot" - realiseerde me dat ik dingen deed die ik niet zelf, actief, bedacht had | | 21,7% |
| Er is iets met mij dat de gebeurtenis deed plaatsvinden | | 21,4% |
| Hoe vaak heeft u gedurende het laatste jaar de behoefte gehad om 's ochtends uw eerste alcoholhoudende drank te gebruiken om weer op gang te kunnen komen na een sessie met overmatig drankgebruik? | | 21,4% |
| Hoeveel glazen alcohol drinkt u op een typische dag wanneer u drinkt? | | 21,4% |
| Bij vlagen had ik er heftige gevoelens over | | 21,4% |
| Ik was gespannen en schrikachtig | | 21,4% |
| Dingen die me eraan herinnerden, ging ik uit de weg | | 21,4% |
| Ik dacht dat ik dood kon gaan | | 21,4% |
| Ik had lichamelijke reacties, zoals zweten, trillen en hartkloppingen | | 21,4% |
| Ik was zeer geschokt door wat ik zag | | 21,4% |
| Ik dacht dat ik mijn emoties niet meer kon beheersen | | 21,4% |
| Ik schaamde me voor mijn emotionele reacties | | 21,4% |
| Ik maakte me bezorgd over mijn eigen veiligheid | | 21,4% |
| Ik voelde me gedesorienteerd, er waren momenten dat ik me onzeker voelde over waar ik was of hoe laat het was | | 21,4% |
| Ik was in de war, er waren momenten dat ik niet goed begreep wat er gebeurde | | 21,4% |
| Er waren momenten dat mijn eigen lichaam als onwerkelijk aanvoelde. Het leek net of ik los was van mijn lichaam, alsof het erg klein of groot was | | 21,4% |
| Ik had af en toe het idee dat ik geen grip meer had over de gebeurtenissen - dat ik een black-out had, in de war was of op de een of andere manier het gevoel had dat ik geen deel uitmaakte van wat er gebeurde | | 21,4% |
| hoeveel herinneren van gebeurtenis | | 21,4% |
| Ik heb geen toekomst | | 21,1% |
| Een ander zou niet in deze situatie terecht zijn gekomen | | 21,1% |
| Mensen zijn niet meer te vertrouwen | | 21,1% |
| Hoe vaak kon u zich in het afgelopen jaar gebeurtenissen van de dag daarvoor niet meer herinneren? | | 21,1% |
| Hoe vaak heeft u gedurende de laatste jaren zich schuldig gevoeld of zelfverwijt gehad over uw drankgebruik? | | 21,1% |
| Hoe vaak heeft u het afgelopen jaar nagelaten om te doen wawt normaal van u werd verwacht vanwege drankgebruik? | | 21,1% |
| Hoe vaak heeft u in het afgelopen jaar opgemerkt dat u niet in staat was het drinken te stoppen madat u was begonnen met drinken? | | 21,1% |
| Ik vermeed erover te praten | | 21,1% |
| Ik droomde erover | | 21,1% |
| Ik probeerde het uit mijn geheugen te bannen | | 21,1% |
| Ik kon moeilijk in slaap komen | | 21,1% |
| Ik vermeed eraan te denken | | 21,1% |
| Het voelde alsof het niet gebeurd was of niet echt was | | 21,1% |
| Als ik eraan herinnerd werd, kwamen de gevoelens terug | | 21,1% |
| Ik dacht dat ik flauw ging vallen | | 21,1% |
| Ik vond het moeilijk om mijn ontlasting en urine op te houden | | 21,1% |
| Ik maakte me zorgen over de veiligheid van anderen | | 21,1% |
| Ik kan niet op andere mensen vertrouwen | | 20,8% |
| Heeft een familielid, vriend, een dokter of een hulpverlener in de gezondheidszorg zijn bezorgdheid geuit over uw drankgebruik en u gesuggereerd uw drankgebruik te minderen? | | 20,8% |
| Heeft u uzelf of iemand anders verwond als gevolg van uw drankgebruik? | | 20,8% |
| Hoe vaak drinkt u alcoholhoudende drank? | | 20,8% |
| Ik verheug me van tevorren al op dingen | | 20,8% |
| Ik voel me alsof alles moeizamer gaat | | 20,8% |
| Als ik eraan herinnerd werd, kreeg ik lichamelijke reacties, zoals zweten, moeilijk ademhalen, misselijkheid of hartkloppingen | | 20,8% |
| Ik kon me moeilijk concentreren | | 20,8% |
| Mijn gevoel erover leek verdoofd | | 20,8% |
| Ik had moeite om 's nachts door te slapen | | 20,8% |
| teveel alledaagse emotionele steun | | 20,5% |
| tekort alledaagse emotionele steun | | 20,5% |
| alledaagse emotionele steun | | 20,5% |
| Hoe vaak drinkt u 6 of meer glazen per gelegenheid? | | 20,5% |
| Ik kan van een goed boek genieten, of van een radios- of tv programma | | 20,5% |
| Ik krijg plotseling gevoelens van panische angst | | 20,5% |
| Ik voel me rusteloos en voel dat ik iets te doen moet hebben | | 20,5% |
| Ik heb geen interesse meer in mijn uiterlijk | | 20,5% |
| Ik krijg een soort benauwd, gespannen gevoel in mijn maag | | 20,5% |
| Ik kan rustig zitten en me ontspannen | | 20,5% |
| ik kan lachen en de dingen van een vrolijke kant zien | | 20,5% |
| teveel informatieves teun | | 20,2% |
| teveel sociale steun | | 20,2% |
| teveel instrumentele steun | | 20,2% |
| teveel waardering | | 20,2% |
| teveel emotionele steun problemen | | 20,2% |
| tekort informatieve steun | | 20,2% |
| tekort sociale steun | | 20,2% |
| tekort instrumentele steun | | 20,2% |
| tekort waardering | | 20,2% |
| tekort emotionele steun problemen | | 20,2% |
| informatieve steun | | 20,2% |
| sociale steun | | 20,2% |
| instrumentele steun | | 20,2% |
| waardering | | 20,2% |
| emotionele steun problemen | | 20,2% |
| Ik voel me opgewekt | | 20,2% |
| Ik maak me vaak ongerust | | 20,2% |
| Ik krijg een soort angstgevoel alsof er elk moment iets vreselijks zal gebeuren | | 20,2% |
| Ik geniet nog steeds van de dingen waar ik vroeger van genoot | | 20,2% |
| Ik voel me gespannen | | 20,2% |
| Sportbeoefening ten tijde T2 | | 17,4% |
| Volledig / deeltijd therap. | | 14,4% |
| Momenteel aan het werk | | 11,0% |
| tijd trauma in uur | | 10,4% |
| psycholoog als behandeling | | 7,6% |
| Tijd in ZH | | 7,0% |
| hospital admission | | 7,0% |
| Verdrietig | | 5,8% |
| Walging | | 5,2% |
| Machteloos | | 5,2% |
| Schuldig | | 5,2% |
| Woedend | | 5,2% |
| Angstig | | 5,2% |
| Bekende overleden | | 4,9% |
| Bekende gewond geraakt | | 4,9% |
| Geconfronteerd met dode personen | | 4,9% |
| Geconfronteerd met gewonde personen | | 4,9% |
| Iemand overleden? | | 4,9% |
| Iemand verwond? | | 4,6% |
| ICU admission | | 4,6% |
| Dacht u dat u dood zou gaan? | | 4,3% |
| 1 = chronisch | | 4,3% |
| Medicatie | | 3,1% |
| Op dit moment last van klachten/stoornis | | 2,8% |
| Aantal uren per week | | 2,8% |
| gewond onderste ledematen | | 2,1% |
| gewond bovenste ledematen | | 2,1% |
| gewond rug | | 2,1% |
| gewond bekken | | 2,1% |
| gewond buik | | 2,1% |
| gewond borst | | 2,1% |
| gewond longen hart borstkas | | 2,1% |
| gewond nek | | 2,1% |
| gewicht gezicht | | 2,1% |
| gewond hoofd | | 2,1% |
| Behandeling | | 2,1% |
| Familie geschiedenis psychische kl./st. | | 1,8% |
| Mijn kind werd seksueel misbruikt | | 1,5% |
| Mijn kind werd lichamelijk mishandeld | | 1,5% |
| ik ben getuige geweest van een moord | | 1,5% |
| opname als behandeling | | 1,5% |
| maatschappelijk werker als behandeling | | 1,5% |
| psychiater als behandeling | | 1,5% |
| Eerdere psychische klachten/stoornis | | 1,5% |
| Mijn kind werd doodgeboren (of miskraam gehad) | | 1,2% |
| Ik ben er getuige van geweest dat iemand fysiek/seks werd mishandeld | | 1,2% |
| Ik ben ernstig sexueel mishandeld geweest | | 1,2% |
| Ik ben ernstig lich. mishandeld geweest | | 1,2% |
| Uur per week aan werk | | 1,2% |
| Werk en dagbesteding | | 1,2% |
| Mijn levenspartner/kind/geliefd persoon is ontvoerd/vermist/gegijzeld geweest | | 0,9% |
| Ik heb iemand tevergeefs gereanimeerd | | 0,9% |
| Ik ben getuige geweest van een zelfmoord | | 0,9% |
| Ik ben ernstig geest. mishandeld geweest | | 0,9% |
| Ik heb een levensbedreigende ziekte (gehad) | | 0,9% |
| Ik heb geassisteerd bij reddingswerkz. na een ramp | | 0,9% |
| Ik heb een ernstig ongeluk meegemaakt | | 0,9% |
| Alles goed herinneren | | 0,9% |
| Thuiswonende kinderen | | 0,9% |
| Ik heb mijn levenspartner/geliefd persoon verloren door een ongeluk/ramp/oorlog/misdrijf/zelfmoord | | 0,6% |
| Ik ben bijna verdronken geweest en moest worden gereanimeerd | | 0,6% |
| Ik heb een oorlog meegemaakt maar niet zelf gestreden | | 0,6% |
| Mijn woning werd vernietigd door een ontploffing/brand/ramp | | 0,6% |
| Aanraking giftige gassen, radioact. straling of ander besmet mat. | | 0,6% |
| Aantal kinderen | | 0,6% |
| Hoogst afgemaakte opleiding | | 0,6% |
| Ik ben bedreigd geweest door iemand of door een groep mensen | | 0,3% |
| Ik heb een oorlog meegemaakt en daarbij zelf gestreden | | 0,3% |
| Ik heb een ramp of grote brand meegemaakt? | | 0,3% |
| Sportbeoefening voor opname | | 0,3% |

| 2-ASAP cohort |  |
| --- | --- |
| Uur per week aan werk | 51,8% |
| tijd trauma in uur | 5,8% |
| hoelang duurde trauma in minuten | 5,6% |
| Volledig of deeltijd aan werk | 3,1% |
| Bekende gewond geraakt | 2,2% |
| medicatie voor eerdere psychische klachten | 2,2% |
| Tijd in ZH | 1,6% |
| Aantal uren per week sport voor trauma | 1,3% |
| Plaats uit ZH | 0,9% |
| Aantal uren per week sport na trauma | 0,4% |
